# Estimating SARS-CoV-2 Seroprevalence in Canadian Blood Donors, April 2020 to March 2021: Improving Accuracy with Multiple Assays

**DOI:** 10.1128/spectrum.02563-21

**Published:** 2022-02-23

**Authors:** Ashleigh R. Tuite, David Fisman, Kento T. Abe, Bhavisha Rathod, Adrian Pasculescu, Karen Colwill, Anne-Claude Gingras, Qi-Long Yi, Sheila F. O’Brien, Steven J. Drews

**Affiliations:** a Dalla Lana School of Public Health, University of Toronto, Toronto, Ontario, Canada; b Centre for Immunization Readiness, Public Health Agency of Canada, Ottawa, Ontario, Canada; c Lunenfeld-Tanenbaum Research Institute at Mt. Sinai Hospital, Sinai Health, Toronto, Ontario, Canada; d Department of Molecular Genetics, University of Toronto, Toronto, Ontario, Canada; e Epidemiology and Surveillance, Canadian Blood Services, Ottawa, Ontario, Canada; f School of Epidemiology and Public Health, University of Ottawa, Ottawa, Ontario, Canada; g Canadian Blood Services, Microbiology, Edmonton, Alberta, Canada; h Department of Laboratory Medicine and Pathology, University of Alberta, Edmonton, Alberta, Canada; University of California, San Diego

**Keywords:** SARS-CoV-2 antibody, spike, receptor binding domain, nucleocapsid, IgG, latent class analysis

## Abstract

We have previously used composite reference standards and latent class analysis (LCA) to evaluate the performance of laboratory assays in the presence of tarnished gold standards. Here, we apply these techniques to repeated, cross-sectional study of Canadian blood donors, whose sera underwent parallel testing with four separate SARS-CoV-2 antibody assays. We designed a repeated cross-sectional design with random cross-sectional sampling of all available retention samples (*n* = 1500/month) for a 12 -month period from April 2020 until March 2021. Each sample was evaluated for SARS-CoV-2 IgG antibodies using four assays an Abbott Architect assay targeting the nucleocapsid antigen (Abbott-NP, Abbott, Chicago IL) and three in-house IgG ELISAs recognizing distinct recombinant viral antigens: full-length spike glycoprotein (Spike), spike glycoprotein receptor binding domain (RBD) and nucleocapsid (NP). We used two analytic approaches to estimate SAR-CoV-2 seroprevalence: a composite reference standard and LCA. Using LCA to estimate true seropositivity status based on the results of the four antibody tests, we estimated that seroprevalence increased from 0.8% (95% CI: 0.5–1.4%) in April 2020 to 6.3% (95% CI: 5.1–7.6%) in March 2021. Our study provides further support for the use of LCA in upcoming public health crises, epidemics, and pandemics when a gold standard assay may not be available or identifiable.

**IMPORTANCE** Here, we describe an approach to estimating seroprevalence in a low prevalence setting when multiple assays are available and yet no known gold standard exists. Because serological studies identify cases through both diagnostic testing and surveillance, and otherwise silent, unrecognized infections, serological data can be used to estimate the true infection fatality ratio of a disease. However, seroprevalence studies rely on assays with imperfect sensitivity and specificity. Seroreversion (loss of antibody response) also occurs over time, and with the advent of vaccination, distinction of antibody response resulting from vaccination as opposed to antibody response due to infection has posed an additional challenge. Our approach indicates that seroprevalence on Canadian blood donors by the end of March 2021was less than 10%. Our study supports the use of latent class analysis in upcoming public health crises, epidemics, and pandemics when a gold standard assay may not be available or identifiable.

## INTRODUCTION

Serological data are often used to profile the change of infectious disease burden over time, and across geographies (1). High-quality seroprevalence data provides several key insights into epidemics: as serology should provide a record of exposure to a given pathogen over time, seroprevalence curves should reveal the true cumulative incidence of disease over the course of an epidemic (2). Because serological studies capture cases identified through diagnostic testing and surveillance, and otherwise silent, unrecognized infections, serological data can be used to estimate the true infection fatality ratio of a disease (3). If seropositivity is a marker for immunity, serological data can also be used to project the likely trajectory of an epidemic, and assess the need for expanded vaccination, as population-level susceptibility is a key determinant of exponential growth of communicable disease epidemics (4).

Estimates of SARS-CoV-2 seroprevalence in Canada during the first quarter of 2021 are impacted by relatively low infections rates and the relatively slow ramp up of vaccination programs (2, 5). Over the study period (April 2020-March 2021), two major pandemic waves occurred in Canada: a spring wave beginning in March 2020 and subsiding in the summer of 2020, and a fall/winter wave. The size of the waves varied substantially across the country, with the Atlantic provinces (New Brunswick, Nova Scotia, Newfoundland and Labrador, and Prince Edward Island) reporting far lower cumulative case rates (<200 cases per 100,000 population) than the other provinces included in this study, which ranged from 1944 per 100,000 in British Columbia to 3354 per 100,000 in Alberta (6). Prior seroprevalence surveys by members of our group has identified very low levels of seropositivity (e.g., <5%) between April 2020 and the second week of January 2021 (*n* = 172,919) in Canadian blood donors (2, 7).

The SARS-CoV-2 vaccine was first administered in Canada during the middle of December 2020 (8). SARS-CoV-2 vaccine supplies were insufficient during the first 3 months of 2021 and Canada would later move to extended dosing intervals for Health Canada approved vaccines (5). Canada adopted a risk- and age-based prioritization approach, with early doses allocated to health care and other front-line workers and residents of long-term care homes and other congregate housing, followed by allocation be descending age groups. By late March 2021, 3.5 million Canadians (9.18% population) had received at least one dose of a SARS-CoV-2 vaccine. Only a small percentage of Canadians had received two doses (1.66% population) (9). Most vaccinated Canadians would have received an mRNA vaccine. The BNT162b2 (Pfizer-BioNTech, New York, NY, USA) mRNA vaccine was received by 6.69% of the population (5.49% received one dose and 1.20% received two doses). The mRNA-1273 (Moderna, Cambridge, MA, USA) was administered to 1.66% of the population (1.21% received one dose and 0.45% received two doses). Few Canadians received a viral vector vaccine with 0.81% of the population receiving one dose of a ChAdOx1-nCOV (Serum Institute of India: Covishield, Pune, India licensed from AstraZeneca, Cambridge, United Kingdom) (9). Before donation, Canadian Blood Services routinely asks donors if they received a SARS-CoV-2 vaccine in the past 3 months. In a prior analysis apart from a “yes” and “no” answer for SARS-CoV-2 vaccination we did not have access to vaccine timing or dose received (10).

During the SARS-CoV-2 pandemic, serological studies have provided a number of key insights into this newly emerged infection; they have affirmed that development of an antibody response correlates with clearance of viral shedding (11); they have helped delineate the fraction of infections that are identified as cases (12); have permitted estimation of infection fatality ratios and have facilitated identification of groups and regions with elevated infection risks and ongoing vulnerabilities to infection (13–16). However, seroprevalence studies rely on assays with imperfect sensitivity and specificity (16). Furthermore, estimation of sensitivity and specificity of these tests is made more challenging by the absence of a gold standard with which they can be compared (2). Seroreversion (loss of antibody response) occurs over time, and with the advent of vaccination, distinction of antibody response resulting from vaccination as opposed to antibody response due to infection has posed an additional challenge (7, 17, 18).

While not perfectly representative of the populations from which they are drawn, blood donors are a subpopulation that allows rapid, repeated evaluation of seroprevalence during a public health crisis (1, 2, 7, 17). Since April 2020 Canadian Blood Services has been performing serological testing on “retention” samples left over from routine testing of donor blood (2, 3, 7, 17). Blood donation occurs in large cities and smaller urban areas. CBS has used several assays in parallel to evaluate SARS-CoV-2 response in donors but has noted that responses of different assays are at times inconsistent with one another (2, 7, 17, 19, 20). Furthermore, we identified differences in serological profiles that could be potentially be linked to variability in humoral protection as measured by antibody neutralizing capacity (19, 21).This led us to ask the question of whether antibody assays can be used in parallel to categorize SARS-CoV-2 immune responses in blood donors.

We have previously used composite reference standards and latent class analysis (LCA) to evaluate the performance of laboratory assays in the absence of agreed-upon gold standards (2, 22). Here, we apply these techniques to repeated, cross-sectional study of Canadian blood donors, whose sera underwent parallel testing with four separate antibody assays. We also attempted a re-linkage of serology data to vaccine dose number and timing of vaccination. Our objectives were (i) to estimate the seroprevalence of SARS-CoV-2 infection in Canadians over the first two waves of the pandemic; (ii) to attempt to define antibody profiles that discriminate prior infection from vaccination (iii) to develop an approach to characterization of population immunity to SARS-CoV-2 that can be reproduced by other groups and with other populations.

## RESULTS

### Study population characteristics.

A total of 17,999 blood donors were included in the study, with samples collected from April 2020 to March 2021. The mean age of participants was 46.3 (standard deviation [SD]: 15.7) years. Participants were more likely to be male (53.9%) and predominantly lived in non-rural locations (87.1%) (Table 1).

**TABLE 1:** Characteristics of *n* = 17999 study^*a*^

Variable	Demographic	Entire study period	April – june 2020	July – september 2020	October – december 2020	January – march 2021	*P* value
	Total participants	17999	4499	4500	4500	4500	
Sex	F	8293 (46.1)^*b*^	2087 (46.4)	2163 (48.1)	2008 (44.6)	2035 (45.2)	0.0058
	M	9706 (53.9)	2412 (53.6)	2337 (51.9)	2492 (55.4)	2465 (54.8)	
Province	Alberta	3715 (20.6)	925 (20.6)	928 (20.6)	941 (20.9)	921 (20.5)	1
	Atlantic provinces	1906 (10.6)	477 (10.6)	478 (10.6)	477 (10.6)	474 (10.5)	
	British Columbia	2743 (15.2)	691 (15.4)	677 (15)	693 (15.4)	682 (15.2)	
	Ontario	7756 (43.1)	1935 (43.0)	1951 (43.4)	1928 (42.8)	1942 (43.2)	
	Manitoba and Saskatchewan	1879 (10.4)	471 (10.5)	466 (10.4)	461 (10.2)	481 (10.7)	
Ethnicity	Aboriginal	213 (1.2)	54 (1.2)	43 (1)	57 (1.3)	59 (1.3)	<0.001
	Asian	708 (3.9)	167 (3.7)	158 (3.5)	187 (4.2)	196 (4.4)	
	White	12954 (72)	3113 (69.2)	3128 (69.5)	3336 (74.1)	3377 (75)	
	Other	1711 (9.5)	360 (8)	403 (9)	508 (11.3)	440 (9.8)	
	Not reported	2413 (13.4)	805 (17.9)	768 (17.1)	412 (9.2)	428 (9.5)	
Age	<40 yrs	6832 (38.0)	1659 (36.9)	1706 (37.9)	1746 (38.8)	1721 (38.2)	0.092
	40−59 yrs	6623 (36.8)	1696 (37.7)	1652 (36.7)	1673 (37.2)	1602 (35.6)	
	60 yrs and over	4544 (25.2)	1144 (25.4)	1142 (25.4)	1081 (24)	1177 (26.2)	
Rural location		2328 (12.9)	545 (12.1)	586 (13)	708 (15.7)	489 (10.9)	<0.001

a *P* values based on chi-squared test.

b No. (%).

### Specimens where vaccination history was available.

Between January and March 2021, 138 of 4500 (3%) study participants reported full or partial vaccination at the time of sample collection. 1.5% (*n* = 2) of vaccinated participants were fully vaccinated (second dose received ≥7 days prior to the date of sample collection); 30.4% (*n* = 42) had received their first dose at least 14 days prior; 31.1% (*n* = 43) received their first dose less than 14 days prior to sample collection; and 37.0% (*n* = 51) of participants reporting having received a least one dose had no information about the timing of dose receipt.

### Overall seroprevalence trends.

**FIG 1:**
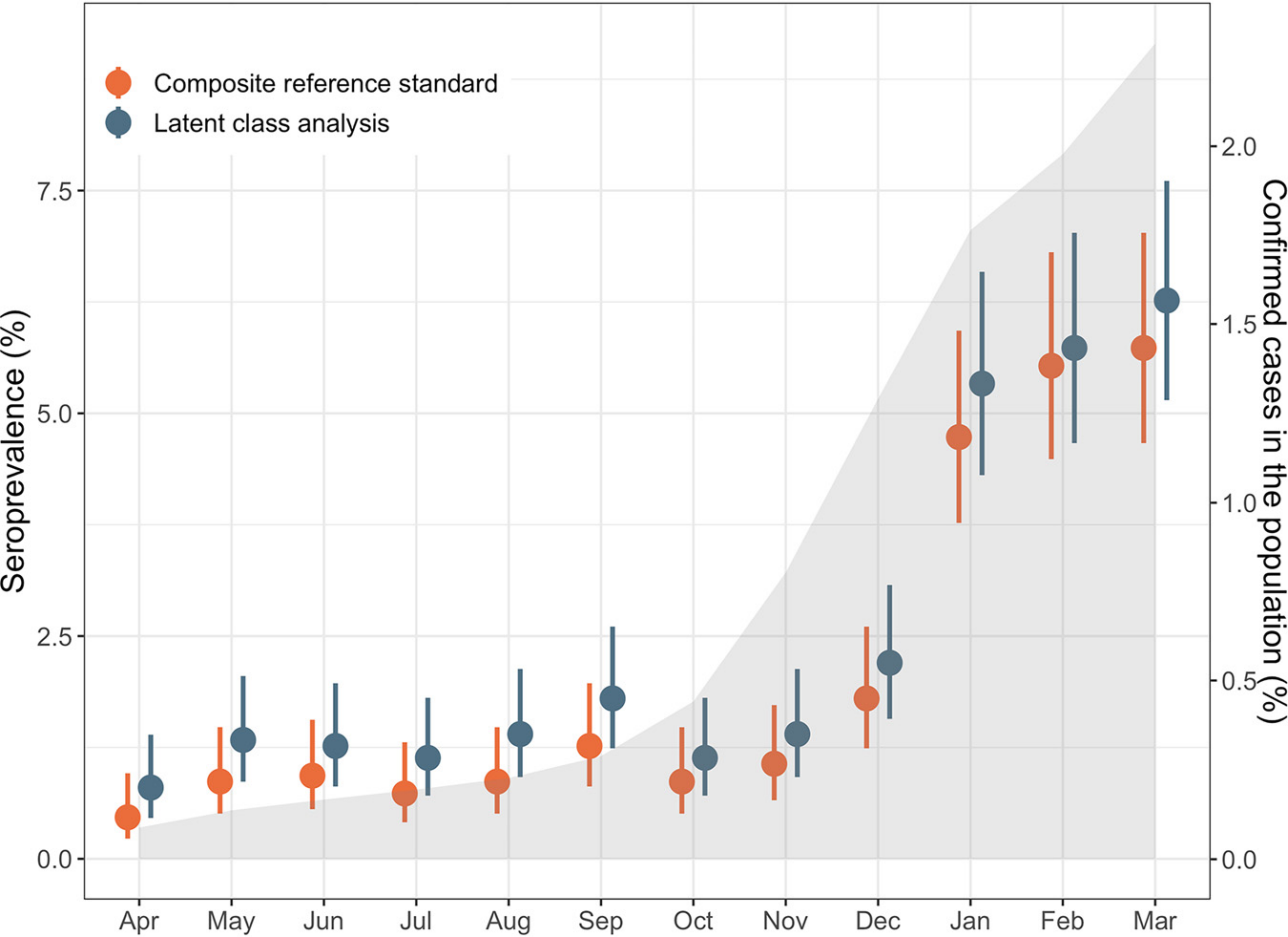
Estimated seroprevalence over time. Estimates were derived using two different approaches, composite reference standard and LCA, as described in the methods. Circles represent mean values and bars indicate the 95% confidence intervals. For comparison, cumulative incidence of laboratory-confirmed SARS-CoV-2 infections in the general population for all provinces excluding Quebec over the study period is shown by the gray shaded area.

**FIG 2:**
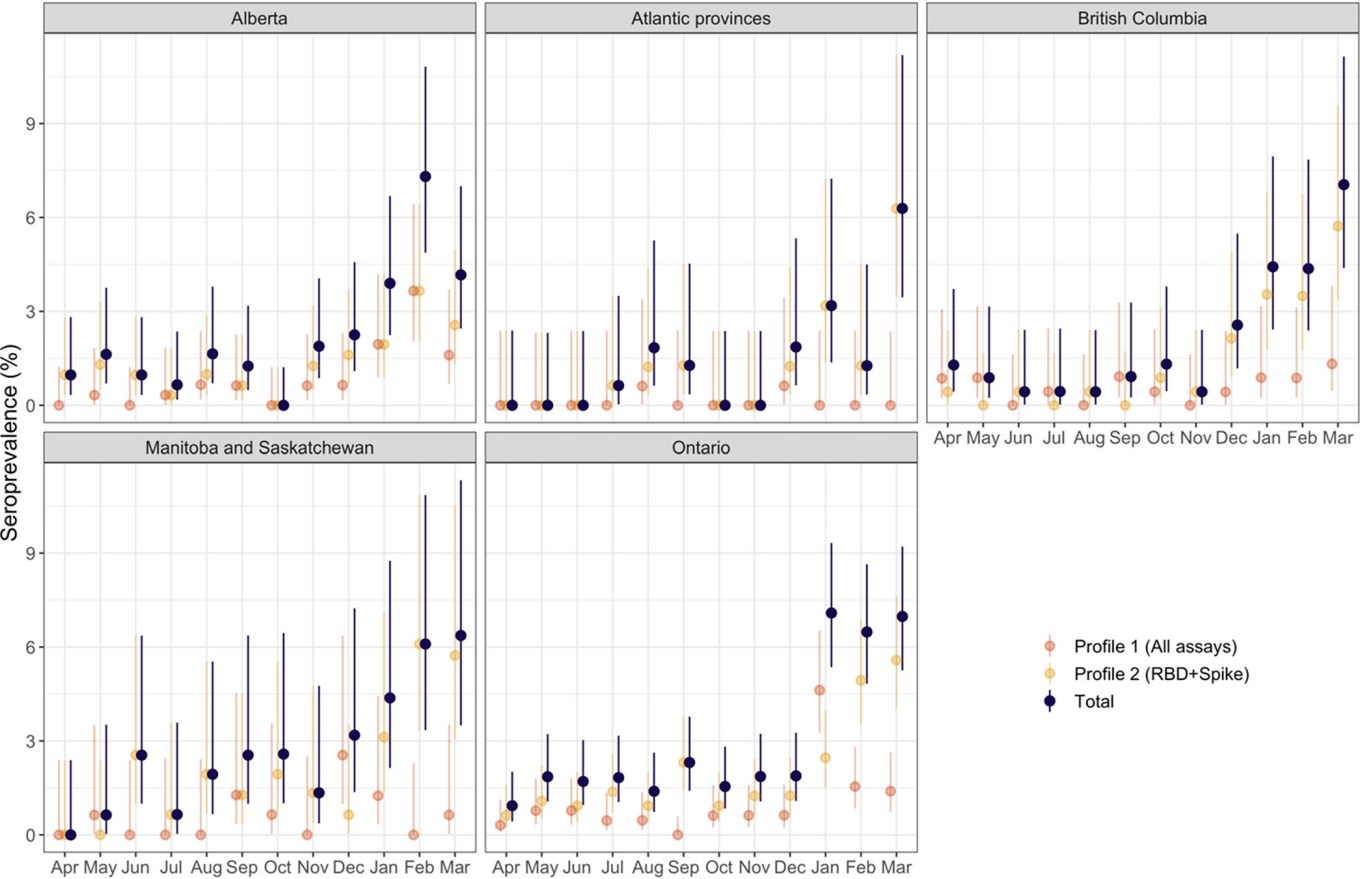
Regional trends in seroprevalence. Seroprevalence estimates are shown for each of the two profiles identified by LCA and the overall results. Circles represent mean values and bars indicate the 95% confidence intervals.

**FIG 3:**
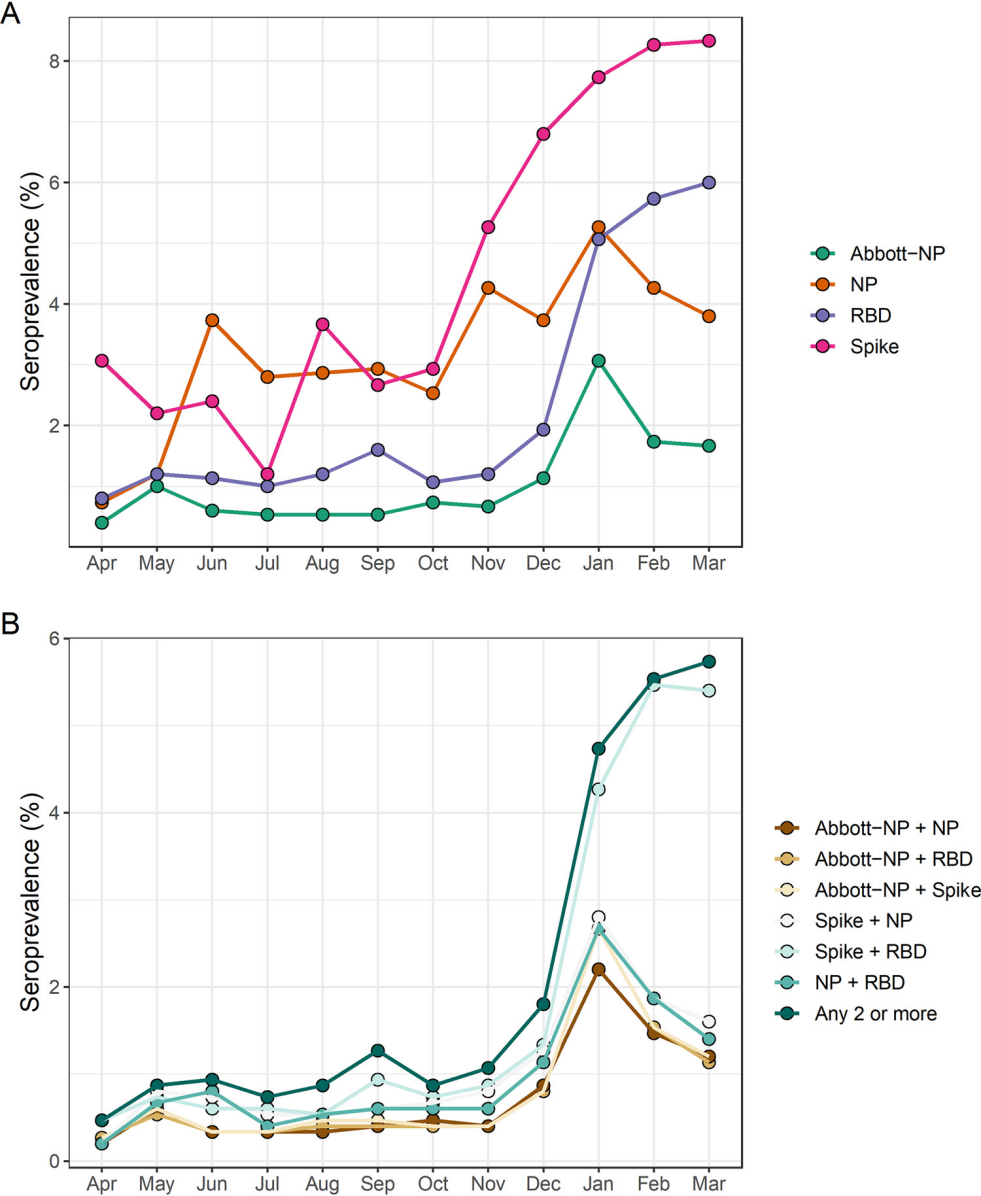
Seropositivity estimates over time based on predefined assay thresholds. (A) Percent of study participants positive for each assay, based on month of sample collection. (B) Percent of study participants positive for two or more assays. The different assay combinations are indicated in the legend and details are provided in the methods.

Using predefined thresholds to determine seropositivity, 92.6% (*n* = 16,667) of study participants were negative for all four assays. 5.2% (*n* = 936) were positive for a single assay only. Based on the assumption that sample seropositivity using two or more assays represents a true positive case, we estimated an overall seroprevalence of 2.1% (95% CI: 1.9–2.3) over the study period. Estimated seroprevalence increased from 0.5% (95% CI: 0.23–0.96%) in April 2020 to 5.7% (95% CI: 4.7–7.0%) in March 2021 using the composite reference standard approach (Fig. 1). Results for the individual assays and different assay combinations are shown in Fig. 2 and 3. Adjusted odd ratios for seropositivity are in Table 2.

**TABLE 2:** Adjusted odds ratios for SARS-CoV-2 seropositivity among study participants

Variable	Demographic	Adjusted odds ratio	95% confidence interval
Sex	F	1 (referent)	
	M	1.16	(0.94–1.44)
Province	Alberta	1 (referent)	
	Atlantic provinces	0.47	(0.28–0.77)
	British Columbia	0.80	(0.55–1.16)
	Ontario	1.21	(0.92–1.6)
	Manitoba and Saskatchewan	1.00	(0.67–1.48)
Time period	April-June 2020	1 (referent)	
	July-September 2020	1.26	(0.87–1.83)
	October-December 2020	1.37	(0.95–1.98)
	January-March 2021	3.18	(2.33–4.43)
Ethnicity	Aboriginal	1.00	(0.35–2.32)
	Asian	1.13	(0.65–1.83)
	White	1 (referent)	
	Other	1.40	(1.01–1.91)
	Not reported	0.96	(0.68–1.34)
Age	<40 yrs	1 (referent)	
	40−59 yrs	0.83	(0.65–1.05)
	60 yrs and over	0.80	(0.6–1.05)
Rural location		1.35	(0.99–1.8)
Vaccinated		94.54	(61.81–148.17)

**FIG 4:**
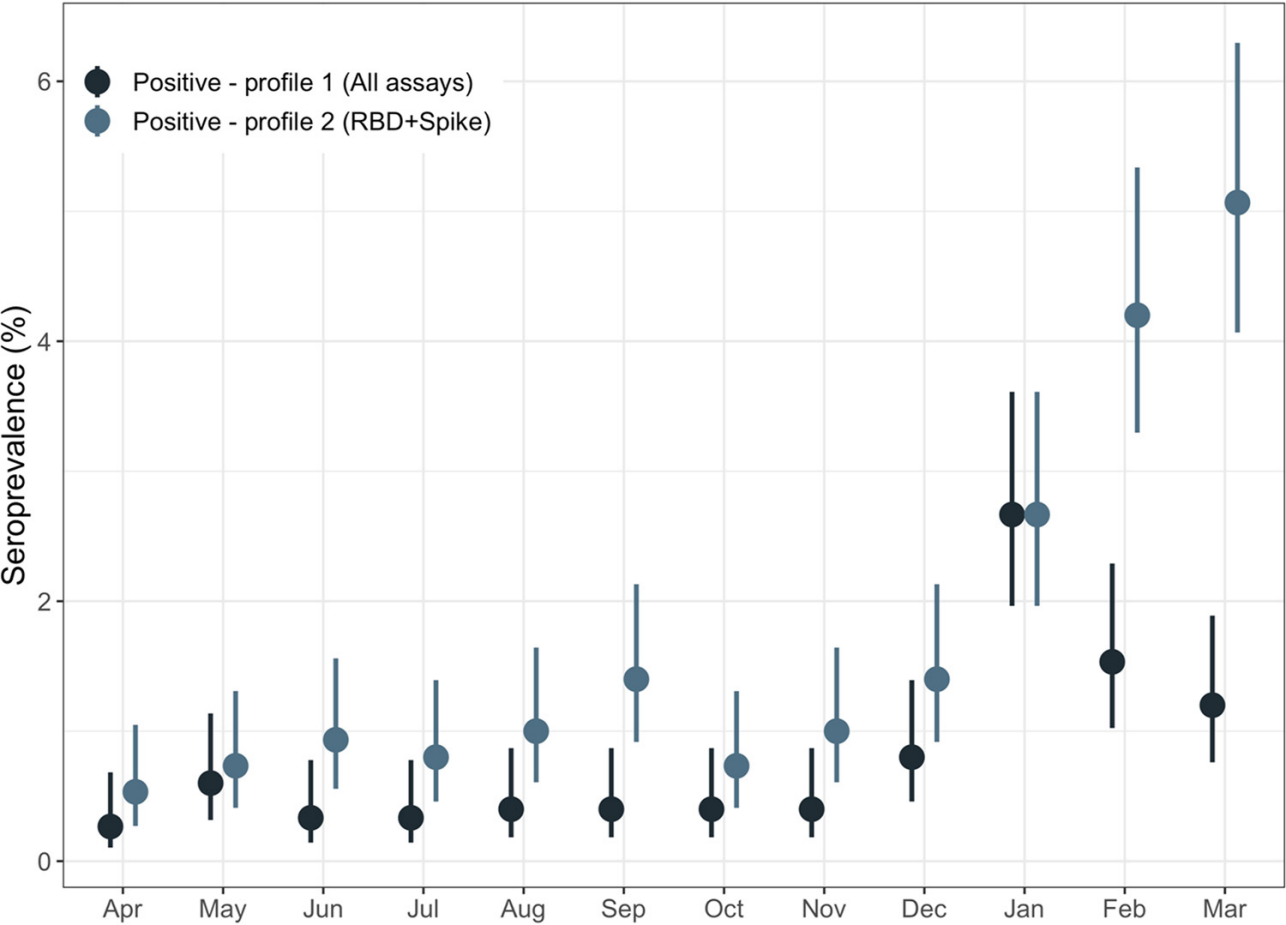
Seroprevalence estimates by latent class grouping. Seropositivity was determined using LCA, with two different classes identified among presumptive seropositive samples. Profile 1 was associated with antibodies to NP, Spike, and RBD, while Profile 2 was associated with antibodies to Spike and RBD.

The best fit LCA model had 3 classes: one class representing the seronegative class, and two different profiles for presumptive seropositive samples (Table 3). For the seropositive classes, one class (profile 1) was associated with antibodies detected in all four assays, while for the other (profile 2), antibodies for RBD and Spike were more discriminatory of seropositivity (Fig. 4).

**TABLE 3:** Group membership probabilities from LCA

Vaccination/infection	Abbott-NP	NP	RBD	Spike
Likely uninfected and unvaccinated	0.003	0.022	0.002	0.021
Profile 1 (likely prior infection)	0.901	0.919	0.976	0.984
Profile 2 (likely vaccinated)	0.020	0.144	0.629	0.768

Using LCA to estimate true seropositivity status based on the results of the four antibody tests, we estimated an overall seroprevalence of 2.5% (95% CI: 2.3–2.7%) for the study period. Seroprevalence increased from 0.8% (95% CI: 0.5–1.4%) in April 2020 to 6.3% (95% CI: 5.1–7.6%) in March 2021 (Fig. 1). Given the strong agreement between the two approaches used to classify seropositivity (Kappa = 0.895, *P* < 0.001), we used the LCA classification results in subsequent analyses.

### Seroprevalence in the vaccinated population.

**FIG 5:**
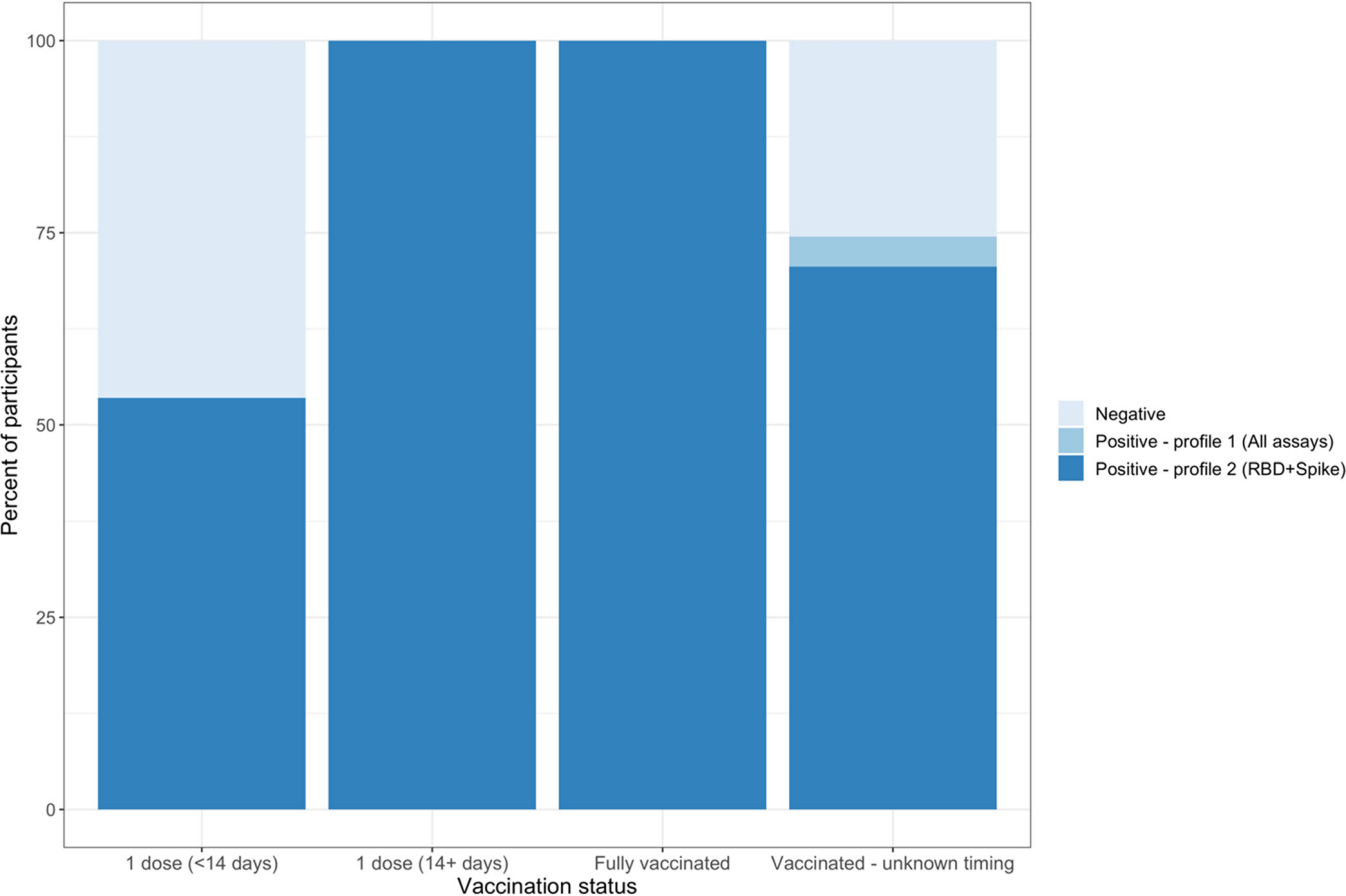
Antibody profiles of vaccinated participants by time since vaccination at time of sample collection. Seropositivity was determined using LCA, with two different classes identified among presumptive seropositive samples. As in Fig. 4, Profile 1 was associated with antibodies to NP, Spike, and RBD, while Profile 2 was associated with antibodies to Spike and RBD.

By LCA, the 44 vaccinated study participants who were either fully vaccinated or had received their first dose at least 14 days prior to sample collection were all classified as profile 2 (positive for Spike and RBD) while those who had received their first dose less than 14 days prior to donation were divided between seronegative (46.5%) and profile 2 (53.5%) (Fig. 5). For the 51 participants with unreported timing of vaccination relative to sample collection, 70.6% had antibody responses suggestive of vaccination-induced seropositivity (profile 2).

### Regional seroprevalence trends.

Estimates of seropositivity by province or region are presented in Fig. 2. Despite regions of the country experiencing very different epidemic trajectories, seroprevalence estimates at the final study time point (March 2021), were not substantially different across the country, ranging from a low of 4.2% (2.5–7.0%) in Alberta to a high of 7.0% (4.4%–11.1%) in British Columbia. There were regional differences in the seropositivity profiles, with the Atlantic provinces dominated by antibody responses suggestive of vaccination (profile 2). In contrast, Alberta, British Columbia, and Ontario saw increases in both types of antibody profiles (profile 1 and profile 2) in the December to March stage of the pandemic. These observations are consistent with the lower rates of infection observed in the Atlantic provinces.

### Factors associated with seropositivity.

After adjustment for age, sex, province, ethnicity, vaccination status, and rural location, the odds of SARS-CoV-2 seropositivity were increased during the January to March 2021 period, relative to April to June 2020 (adjusted odds ratio (OR): 3.18, 95% confidence interval 95% CI: 2.33–4.43) (Table 2). Residence in the Atlantic provinces was protective (OR: 0.47, 95% CI: 0.28–0.77), while self-reported ethnicity of “Other” was associated with increased risk of being seropositive (OR: 1.40, 95% CI: 1.01–1.91). As expected, being vaccinated was strongly associated with seropositivity.

## DISCUSSION

SARS-CoV-2 seropositivity was low but increased over the first year of the pandemic in a sample of Canadian blood donors. This is supported by our earlier work describing this low level of seroprevalence (2, 19). Given the absence of a gold standard (2, 16), we used two approaches to estimate SARS-CoV-2 seropositivity, and results were consistent across methodologies. We also attempted to understand the impact of donor declared vaccine history on SARS-CoV-2 serological profiles. This study did not attempt to infer neutralizing antibody seroprotection from the seroprevalence estimates, as we have previously noted that individuals with anti-S and anti-RBD antibodies may have significant variability in neutralizing capacity against wild type and variant SARS-CoV-2 (19–21).

In LCA, we identified two subgroups of seropositive cases. The first profile (profile 1), with antibodies detected in all four assays used, would be consistent with natural infections. It is possible that some people in this group were vaccinated but had either false-positive results (we feel this is less likely) for nucleocapsid or a positive signal for nucleocapsid due to cross-reactivity with seasonal coronaviruses. The second profile (profile 2), with RBD and Spike antibodies more predictive of seropositivity, would be expected in vaccinated people or potentially in people with a prior infection where nucleocapsid antibodies have waned (7, 17). Among people with known vaccination status, test positivity profiles for RBD and Spike antibodies were consistent with expectation, but it is important to note that the lack of nucleocapsid may not be precise for classifying vaccine status, and some vaccinated people may also have been previously infected (1, 14).

From April to December 2020, estimated seroprevalence was relatively flat, though there was some regional variability in trends. This trend aligns with our previous studies (2, 7, 17). Between December 2020 and January 2021, we observed a marked 2.5-fold increase in seropositivity, from approximately 2% to 5%. This inflection point occurred at a point in time when vaccination program rollout had begun across the country and when the second wave was reaching its peak (23). While we noted an overall increase in seroprevalence, there was an apparent decline in seropositivity associated with nucleocapsid, a marker of natural infection. The reason for this decline is uncertain but could represent antibody waning for people infected in the first pandemic wave or those who were prioritized for vaccination (April to July 2020) (7, 8, 17).

This study used a relatively small sample population (*n* = 17,999) collected over a 12-month period to understand the complexities of anti-SARS-CoV-2 assays and undertake parallel testing. Other surosurveys of healthy Canadians have identified that the combination of natural and vaccine mediated anti-SARS-CoV-2 was less than 10% in the first 3–4 months of 2021. In a larger monthly sampling of Canadian blood donors by Canadian Blood Services and the Canadian Immunity Task Force tested 16,873 specimens by both the Roche semiquantative anti-S and qualitative anti-N assays (Indianapolis, IN) (February 27-March 13, 2021). In that unpublished study, an adjusted anti-S seroprevalence of 9.9% estimated while an adjusted anti-N of 3.3% was estimated (24). A Statistics Canada and Canadian Immunity Task Force-led dried blood spot survey tested approximately 11,000 Canadians aged 1 and older living in private households between November 2020 to April 2021. Using an aggregate standard of the same immunoassays (except Abbott-anti-N) used in our study, a seroprevalence estimate for Canadian adults aged 20 to 59 by April 2021 was estimated at 4.5%. For Canadians aged 60 and older, the overall antibody seroprevalence was 2.1% (25, 26). It is possible that differences in Canadian seroprevalence rates between studies may be due to a variety of factors, including sample size, study population, antigen detected, assay used and determinant of seropositivity used. Regardless of the approach, in March of 2021, infection and vaccine-mediated seroprevalence in Canadian blood donors (<10%) was much lower than US seroprevalence estimates. At that time, seroprevalence rates for US blood donors were approximately 20% for natural infection and approximately 50% for combined infection and vaccine-mediated seropositivity (1).

Although we did not identify substantial differences in overall seropositivity estimates across the country, we did identify regional differences in the seropositivity profiles, with the Atlantic provinces dominated by antibody responses suggestive of vaccination. In contrast, Odds Ratios for seropositivity were highest in Ontario followed by Alberta (referent), Saskatchewan/Manitoba (combined) and then British Columbia in the December to March stage of the pandemic. These observations suggest lower rates of infection observed in the Atlantic provinces. Our regional results are slightly different than the Statistics Canada and Canadian Immunity Task Force-led dried blood spot survey. That survey suggested that SARS-CoV-2 seroprevalence due to a past infection was higher in Alberta (4.0%), followed by Saskatchewan (2.9%), Ontario (2.5%), Manitoba (2.4%) and British Columbia (1.6%). However, similar to our study, the Atlantic region had the lowest seroprevalence due to past infection (25). Differences between the two studies may be due to a variety of factors as described earlier but also might be due to inclusion of April data in the other study would capture third wave cases (27).

This study has several caveats. We utilized a relatively small number of specimens over a 12-month period. This may create some small sample size driven biases when estimating regional seroprevalence rates. The methodologies used to detect antibody were qualitative and we did not analyze the difference in changing antibody titers over time. We also utilized donor-declared vaccine histories which often lacked information such as dose received and timing of the vaccine dose. There was no information available in the donor records on the type of vaccine (mRNA, brand, vector-based) received. The study was also undertaken as variants of concern were emerging and there was an increasing proportion of N501Y VOCs (e.g., Alpha, Beta and Gamma) identified between mid February and late March 2021 (28). Antibody profiles in our study may differ from those in the late summer of 2021 were Delta VOCs (29) and almost all Canadian Blood donors were anti-S positive due to vaccination (30).

In conclusion, we describe an approach to estimating seroprevalence in a low prevalence setting when multiple assays are available and yet no known gold standard exists. Our approach indicates that seroprevalence on Canadian blood donors by the end of March 2021 was less than 10%. Here, we show a largely susceptible population prior to the large scale roll out of SARS-CoV-2 vaccines. We also show some regional variabilities in blood donor seroprevalence rates. Estimating seroprevalence rates in low prevalence settings can be difficult and confusing. However, our study provides further support for the use of LCA (2, 22) in upcoming public health crises, epidemics, and pandemics when a gold standard assay may not be available or identifiable. In the future, we intend to analyze later subsets of donations with respect Delta and Omicron variants of SARS-CoV-2.

## MATERIALS AND METHODS

### Ethical considerations.

This project received ethics board clearance from the following institutions Canadian Blood Services, the University of Alberta and Sinai Health, Toronto (Lunenfeld-Tanenbaum Research Institute).

### CIHR correlates of immunity study participants and samples.

Canadian Blood Services has blood collection sites concentrated in large and small cities in all Canadian provinces except Quebec. Blood donors must meet the following criteria: be at least 17 years of age; pass health selection criteria screening and pass infectious diseases screening protocols for blood donations that are then used to manufacture products for transfusion. At each donation, there is also an additional EDTA plasma (Becton Dickson [BD], Mississauga, ON, Canada) retention sample collected for additional blood testing if required (31).

### Collection of SARS-CoV-2 vaccination history in donors.

All donors at the time of donation were asked if they received a SARS-CoV-2 vaccine in the past 3 months. This was standard practice by Canadian Blood Services. Prior to this study, the study group had access to “yes” and “no” answers for vaccine history. In the autumn of 2021, a relinkage of specimen data to any vaccine history was attempted by Canada Blood Services database managers outside this study group. This also meant that information on the source of vaccine (e.g., producer) was not collected as this was not collected at the time of donation. Provincial vaccine databases are not linked to the blood operator records of donation.

### Study design and population.

We designed a repeated cross-sectional design with random cross-sectional sampling of all available retention samples (*n* = 1500/month) for a 12 -month period from April 2020 until March 2021. A two-stage process sampling approach was used with a random selection of blood donor clinics followed by a random sample selection within clinics. Samples were anonymized. We collected variables, including sex, birth year, residential Forward Sortation Area (FSA, first three characters of postal code), donation date, and collection site were extracted from the Canadian Blood Services donor database. Retention plasma specimens were aliquoted at Canadian Blood Services and transported to test sites (2). One aliquot (250 μl) was stored at −80°C for the remainder of the study.

### Comparison of seroprevalence estimates to observed trends in reported infections.

To compare seroprevalence estimates to observed trends in reported infections, we obtained data on cumulative incidence of laboratory-confirmed SARS-CoV-2 infections for all provinces excluding Quebec from (6). Note that these data included reported infections only, were collected over a time period with various criteria for accessing testing and were not restricted by age.

### SARS-CoV-2 antibody testing.

Each retained plasma sample was evaluated for SARS-CoV-2 IgG antibodies using four assays, as described previously (2). This study used an Abbott Architect anti-nucleocapsid antigen assay (Abbott-NP, Abbott, Chicago IL) as well as three in-house IgG ELISAs utilizing recombinant viral antigens: full-length spike glycoprotein (Spike), spike glycoprotein receptor binding domain (RBD) and nucleocapsid (NP) (32, 33).

Specimens used for this study included those described in a prior analysis for April-September 2020, inclusive (2). Those results were included in this study and reanalyzed using a different approach as described below.

### Estimation of seropositivity status and seroprevalence in the study population.

For each assay, the threshold for positivity was determined by defined cutoffs provided either by the assay manufacturer (1.4 for Abbott-NP) or based on 3 standard deviations from the log mean values of negative controls for the in-house assays (0.19 for spike, 0.186 for RBD, and 0.396 for NP) (2, 32). In the absence of a gold standard test, we used two analytic approaches to estimate SAR-CoV-2 seroprevalence: a composite reference standard and LCA.

Using the composite reference standard approach, a sample was classified as a “true” positive if it was positive for two or more of the assays. LCA is form of latent variable modeling that focuses on identifying subpopulations or groups within a sample based on a certain set of variables and has been used in infectious disease diagnostics to determine positivity when multiple imperfect tests are available (22). In this analysis, the latent variable of interest was infection with SARS-CoV-2 or vaccination against SARS-CoV-2. Based on the four assays (indicator variables), individuals from our sample population are assigned (with a certain probability) to one of two classes: seropositive or seronegative.

Given that our sample includes vaccinated people, who are expected to be positive for spike and RBD but not nucleocapsid, and some infected people may have experienced antibody waning in the time between infection and sampling, we explored models that allowed up to 4 distinct profiles (that is, we assumed that positive samples could have more than one distinct profile across the four assays) and assumed each assay was independent of the others, conditional on a person’s antibody status. Models were estimated using Gibbs sampling, with a burn-in of 1000, 10,000 iterations, and uniform priors. Fit was assessed using the Bayesian Information Criterion (BIC). We used our best-fit model to predict the true seropositivity status for each participant and used this classification to estimate seroprevalence in the study population over time.

We used Wilson’s method to calculate 95% confidence intervals (CIs) and Cohen’s kappa to compare agreement between the two methods used to classify seropositivity. We also constructed a logistic regression model to examine associations between seropositivity and age, sex, and geography, and other factors.

### Data storage and statistical analysis.

Microsoft Excel (Redmond, WA, USA) spreadsheet was used for data storage. Data were analyzed as described in the results section using. All data analysis and statistics was performed using R (34, 35).
